# Age-dependent effects of blue light exposure on lifespan, neurodegeneration, and mitochondria physiology in *Drosophila melanogaster*

**DOI:** 10.1038/s41514-022-00092-z

**Published:** 2022-07-27

**Authors:** Yujuan Song, Jun Yang, Alexander D. Law, David A. Hendrix, Doris Kretzschmar, Matthew Robinson, Jadwiga M. Giebultowicz

**Affiliations:** 1 Department of Integrative Biology, Oregon State University, Corvallis, OR 97331 USA; 2 Department of Biochemistry and Biophysics, School of Electrical Engineering and Computer Science, Corvallis, OR 97331 USA; 3 Oregon Institute of Occupational Health Sciences, Oregon Health and Science University, Portland, OR 97239 USA; 4 School of Biological and Population Health Sciences, College of Public Health and Human Sciences, Oregon State University, Corvallis, OR 97331 USA

**Keywords:** Ageing, Risk factors

## Abstract

Blue light is a predominant component of light emitting devices (LEDs), which are increasingly present in our environment. There is already accumulating evidence that blue light exposure causes damage to retinal cells in vitro and in vivo; however, much less is known about potential effects of blue light on non-retinal cells. That blue light may be detrimental at the organismal level independent from retinal effect was recently shown by findings that it reduces lifespan in worms and also in flies with genetically ablated retinas. Here, we investigated the effects of blue light exposure across the fly lifespan and found that susceptibility to blue light stress is strongly age-dependent. The blue light of the same intensity and duration reduced survival and increased neurodegeneration more significantly in old flies than in young flies. These differences appear to be caused, at least in part, by impairments of mitochondrial respiratory function. We report that blue light significantly reduces the activity of Complex II in the electron transport system and decrease the biochemical activity of succinate dehydrogenase in both young and old flies. In addition, complex I and complex IV activities are reduced by age, as are ATP levels. We therefore propose that older flies are more sensitive to blue light because the light-induced mitochondrial damage potentiates the age-related impairments in energy metabolism that occurs even in darkness. Taken together, our results show that damaging effects of blue light at the organismal level are strongly age dependent and are associated with reduced activity of specific components of energy producing pathways in mitochondria.

## Introduction

Blue light (BL) is becoming increasingly prevalent in our environment raising concerns about its safety^1,2^. In fact, research on cells and model organisms suggests that BL may have a range of detrimental effects. A single acute blue light exposure causes photoreceptor death in the retina of mice and *Drosophila*^3,4^. In addition, both retina-derived and non-retinal cells cultured in vitro produce reactive oxygen species (ROS) and may undergo apoptosis depending on blue light intensity and exposure time^5,6^. At the organismal level, visible light, especially the blue part of the spectrum, causes oxidative stress and shortens the lifespan of the nematode *C. elegans*^7^. We previously reported that adult *Drosophila* maintained in cycles of 12 h BL and 12 h darkness show symptoms of accelerated aging, such as impaired locomotor performance, brain neurodegeneration, and reduced lifespan compared to flies reared in light with blue wavelengths filtered out, or in constant darkness^8^. In addition, BL caused the death of photoreceptor cells; however, lifespan shortening did not depend on the photoreceptor death, as it was also observed in BL-exposed flies with genetically ablated eyes, suggesting that BL is damaging to non-retinal cells^8^.

In general, aging is accompanied by a decline in tolerance to environmental stresses. Blue light-enriched LED is a relatively new factor in our homes and workspaces; therefore, it is unknown whether chronic BL exposure across organismal lifespan could be detrimental to cellular functions and whether susceptibility to BL may change with age. Here, we addressed these questions in a short-lived model organism, *Drosophila melanogaster*. To explore responses to BL across the lifespan, we measured the survival rate of flies that were kept in constant darkness (DD) and then transferred to constant BL at a progressively older age. We report that aging flies become more susceptible to BL as demonstrated by the reduced survival and increased neurodegeneration. To investigate the causes of age-related light sensitivity, we focused on mitochondria and found light-dependent and age-dependent impairments in mitochondrial respiratory function that together underlie increased phototoxic effects of BL in older flies.

## Results

### Aging flies show reduced survival and increased neurodegeneration under constant blue light

**Fig. 1:**
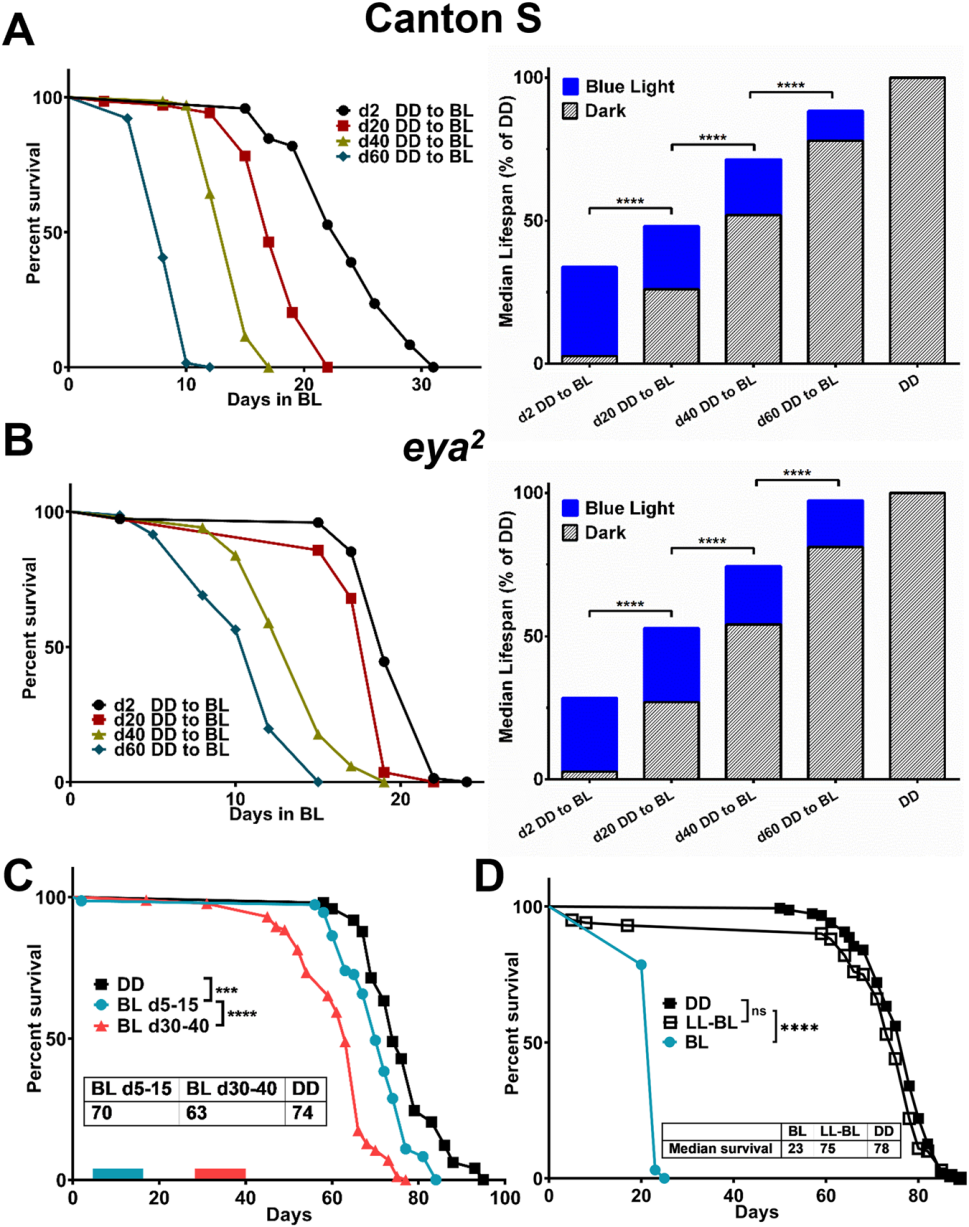
Susceptibility to blue light increases with age. Mortality was recorded for (**A**) Canton S (CS) and (**B**) *eya*^*2*^ males transferred from darkness (DD) to blue light (BL) at progressively older age indicated in the graph legend. Graphs on the left show mortality curves starting from the day when flies were transferred from DD to BL. Graphs on the right show median survival in BL added to days in DD with survival of flies raised in DD shown as reference. Stars indicate significant differences in survival in BL based on statistics by log-rank test (****p < 0.0001). (**C**) Lifespan of flies kept in DD or exposed to BL for 10 days, from 5- to 15-day old (blue bar on the *x*-axis) or from 30- to 40-day old (red bar on the *x*-axis). **D** Lifespan was measured in flies kept in constant blue LED light or constant white LED light with blue wavelengths blocked by a yellow filter (LL-BL). Statistics for mortality curves by log-rank test (***p < 0.001 ****p < 0.0001).

To investigate responses to BL in flies across their lifespan, we tested survival of flies that were kept in DD and then transferred to constant BL when 2-, 20-, 40-, or 60-day old. Mortality curves compared by log-rank test show that the survival of wild type Canton S (CS) males in BL is significantly reduced proportionally to the age at which they were transferred to BL conditions (Fig. 1A). Given previous findings that the compound eyes are damaged by BL^4,8^, we conducted similar experiment in *eya*^*2*^ mutants with genetically ablated eyes^9^ to exclude effects caused by retinal degeneration. While completely missing compound eyes, these flies retain two small visual organs, the ocelli and the Hofbauer–Buchner eyelets^10^. Similar to CS flies, the lifespan of *eya*^*2*^ males in BL was also shortened proportionally to the age at which the flies were transferred from DD to BL (Fig. 1B), showing that the lifespan reduction is independent of the retinal damage. Together, these data show that young CS and *eya*^*2*^ flies can survive in BL 2-3 times longer than old flies, suggesting that the flies become more susceptible to BL damage as they age. As our aim was to understand BL effects on extra-retinal cells, all subsequent experiments were conducted in *eya*^*2*^ flies.

We next tested whether exposure of young (day5) and middle-aged (day30) *eya*^*2*^ males to 10 days in BL affected their overall longevity when returned to darkness. The lifespan of flies exposed to BL even at the young age was significantly reduced (by 6%) compared to the control in DD (*p* < 0.001; Fig. 1C). Moreover, the lifespan of flies exposed to the same BL conditions at the middle age was further reduced by 15% compared to DD control and was significantly shorter (*p* < 0.0001) compared to flies exposed to10 days of BL at the young age (Fig. 1C). Taken together, these data suggest that exposure of flies to non-lethal BL has long-term health consequences persisting beyond the period of exposure. To verify that observed effects are caused specifically by blue light, we exposed *eya*^*2*^ males to constant BL or constant white light of the same irradiance with blue wavelengths filtered out. Consistent with our previous report^8^, survival of flies exposed to light missing blue part of the spectrum was not significantly different from flies kept in DD (Fig. 1D).

**Fig. 2:**
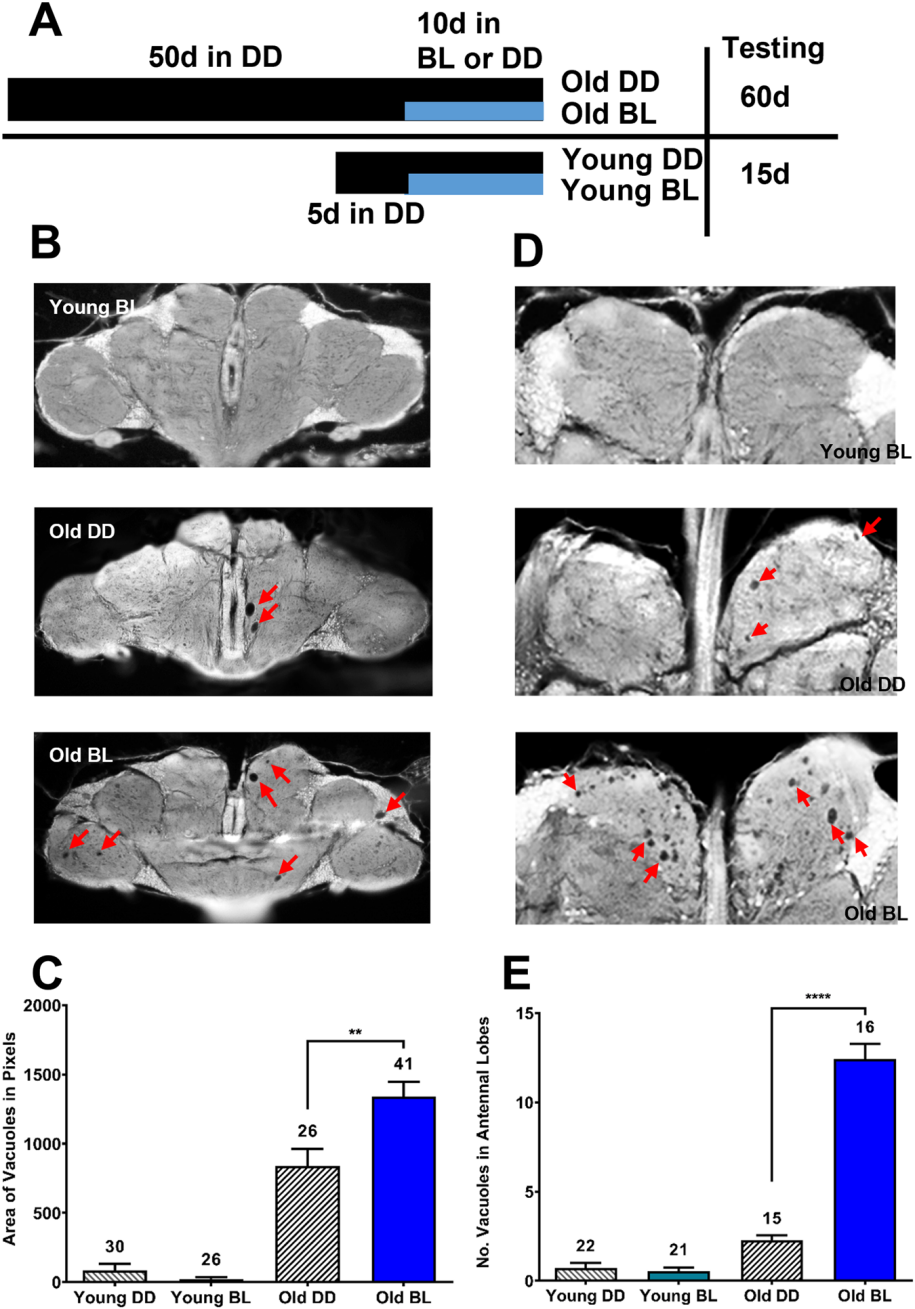
Blue light exposure leads to increased neurodegeneration in the aging fly brain. **A** Scheme of the experimental design showing the treatment of flies before they were fixed to measure brain neurodegeneration. **B** Representative images of brain sections showing brain vacuoles (red arrows) in young BL, old DD, and old BL flies. **C** Quantification of the average area of vacuoles. **D** Images of antennal lobes in young BL, old DD, and old BL flies with small vacuoles marked by red arrows. **E** Quantification of the average number of vacuoles in the antennal lobes. Statistics in (**C**) and (**E**) by unpaired *t* test (**p < 0.01, ****p < 0.0001). Numbers above bars indicate the sample size for each age and light condition. Error bars indicate standard error of the mean (SEM).

Given that flies become more susceptible to blue light as they age, we investigated whether this may be due to increased neuronal damage. We evaluated brain neurodegeneration in young and old *eya*^*2*^ males after they were exposed to BL for 10 days compared to flies kept in the dark for the same number of days (see scheme in Fig. 2A). For this purpose, heads were sectioned to measure the size of vacuoles indicative of brain cell loss. Young flies kept in DD or BL displayed minimal vacuolization with no significant differences between the conditions (Fig. 2B, C). In contrast, a significant increase in brain vacuolization was detected in 60-day-old flies that were exposed to BL for 10 days compared to age-matched DD controls (Fig. 2B, C). Additional analysis of brain sections at the level of the antennal lobes revealed numerous smaller vacuoles, especially prominent in old flies exposed to BL (Fig. 2D). Quantification of the vacuoles in the antennal lobes showed a significant increase (*p* < 0.0001) after BL exposure in old but not young flies compared to age-matched controls in DD (Fig. 2E). Thus, while BL did not affect brain vacuolization in young flies, exposure of old flies to BL of the same length and intensity significantly increased brain neurodegeneration.

### Respiration of mitochondria is impaired differently by blue light and age

**Fig. 3:**
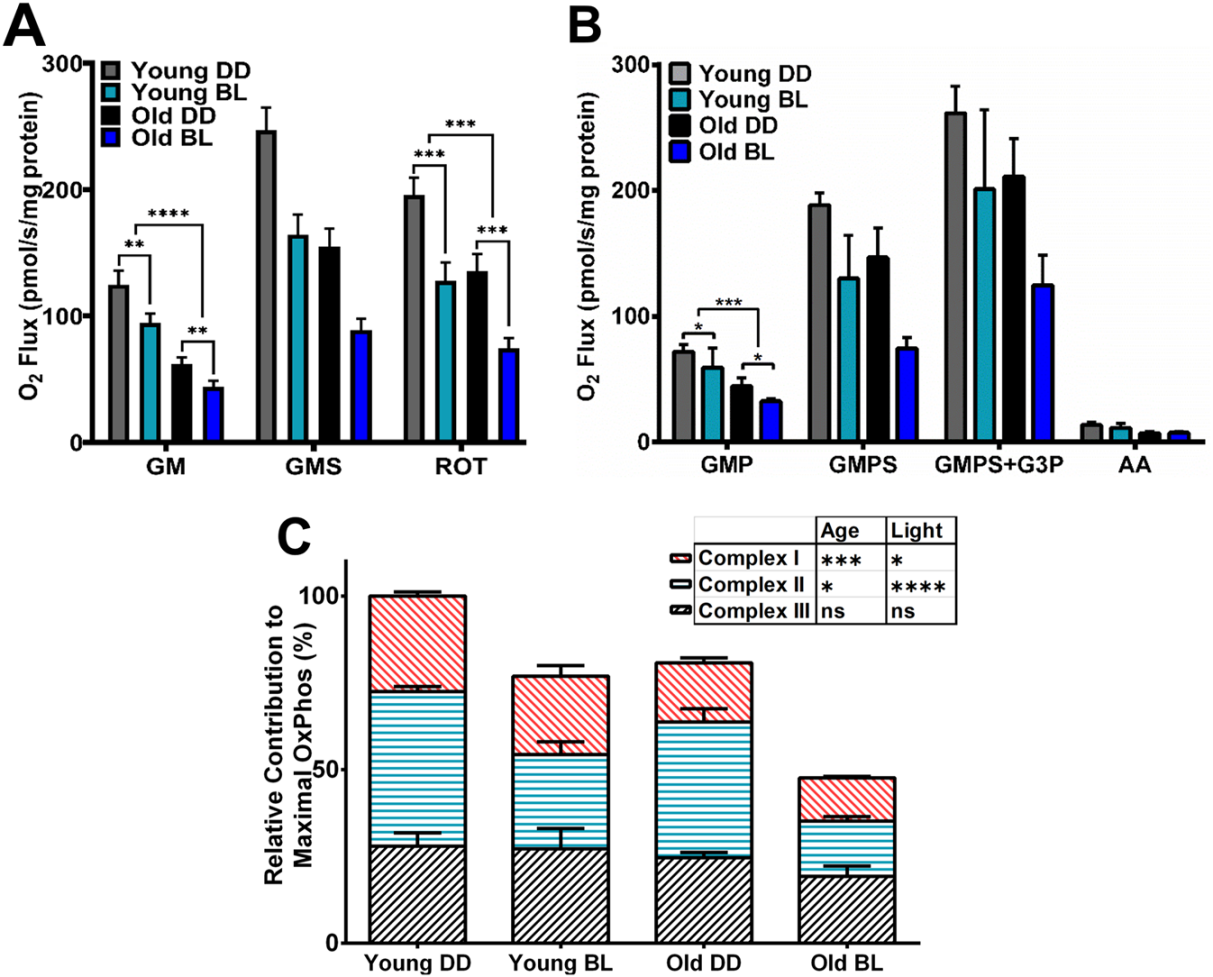
Respiratory functions of mitochondria are affected by both BL exposure and age. **A** Oxygen consumption by CI after addition of ADP, glutamate and malate (GM), CI + CII after addition of Succinate (GMS) and complex II alone after addition of the CI inhibitor rotenone (ROT). *N* = 6 independent biorepeats. Error bars indicate standard error of the mean (SEM). **B** Oxygen consumption by CI after addition of ADP, glutamate, malate, and pyruvate (GMP), CI + CII after addition of succinate (GMPS), CI + CII + G3P after addition of glycerol-3-phosphate (G3P), and residual oxygen consumption after inhibiting mitochondria with antimycin A (AA). *N* = 4 independent biorepeats. Error bars indicate SEM. **C** Data from Fig. 3B were calculated as a relative contribution of CI, CII, and CIII to total respiration, which is set as 100% for the young DD control. Statistical analysis by 2-way ANOVA, (*p < 0.05, ***p < 0.001, ****p < 0.0001). Error bars indicate SEM.

It has been hypothesized that BL may negatively affect mitochondria^11^; however, effects of BL on mitochondrial respiration were not directly investigated. Here, we compared oxygen consumption in mitochondria obtained from young and old *eya*^*2*^ males exposed for 10 days to continuous BL with control males of the same age maintained in DD (see experimental scheme in Fig. 2A). Oxygen flux (*J*O_2_) was measured at 25 °C in mitochondria prepared from whole flies in MIR05 respiration buffer using high-resolution respirometry Oxygraph-O2K. The mitochondria were added to the chamber, and the respiration rates for Complex I (CI) were obtained under phosphorylating conditions after injection of ADP followed by glutamate and malate (GM). We used several respiration protocols that separated the individual input through specific complexes (to determine complex specificity) versus the combined input of several substrates. Together, these analyses determine impact of age and BL at individual and combined activity of respiratory complexes. Oxygen consumption by CI was significantly lower (*p* < 0.0001) with age in both DD and BL flies and was reduced to a lesser degree (*p* < 0.01) by BL in both young and old flies (Fig. 3A). Next, Complex II (CII) activity was measured after the addition of succinate (GMS) and subsequent inhibition of CI by rotenone (ROT). Both young and old flies exposed to BL showed dramatically decreased CII activity (*p* < 0.001) compared to their age-matched DD controls; in addition, CII activity was significantly decreased by age (Fig. 3A).

In a follow-up experiment, we also supplied pyruvate as the third substrate for CI in addition to glutamate and malate (GMP). Providing three substrates avoids the possibility that lower respiration is due to a specific substrate but instead due to inhibition of complex I. The addition did not substantially change relative O_2_ flux through CI indicating complex I activity was maximized, thus the lower respiration of complex I was not specific to the substrate (Fig. 3B). Subsequent addition of succinate (GMPS) confirmed the reduced activity of CII in the presence of BL in both young and old flies (Fig. 3B). In addition to CI and CII, we probed activity of the glycerophosphate dehydrogenase complex, which transfers electrons to ubiquinone through Complex III (CIII), by injecting glycerol-3-phosphate (G3P), which gives a measure of O_2_ flux in addition to CI and CII. The addition of G3P increased respiration rates over complex I and II in all conditions tested but respiration remained lower in BL and with aging.

To better compare and visualize the substrate titration data, we calculated and plotted the relative contribution of CI, CII, and CIII to overall oxidative phosphorylation based on data from Fig. 3B. The maximal respiration in young DD flies was set as 100% and then we calculated relative respiration by each complex. As shown in Fig. 3C, CI activity is impaired more significantly by age than by BL and the reverse is true for CII. Respiration of G3P, which does not require CI or CII, was not affected significantly by either age or BL conditions. Overall, BL treatment reduced the maximal respiration by ~25% in young flies and by ~40% in old flies compared to age-matched DD controls. (See Fig. 6 for schematic summary of these changes).

### Biochemical characterization of mitochondria

**Fig. 4:**
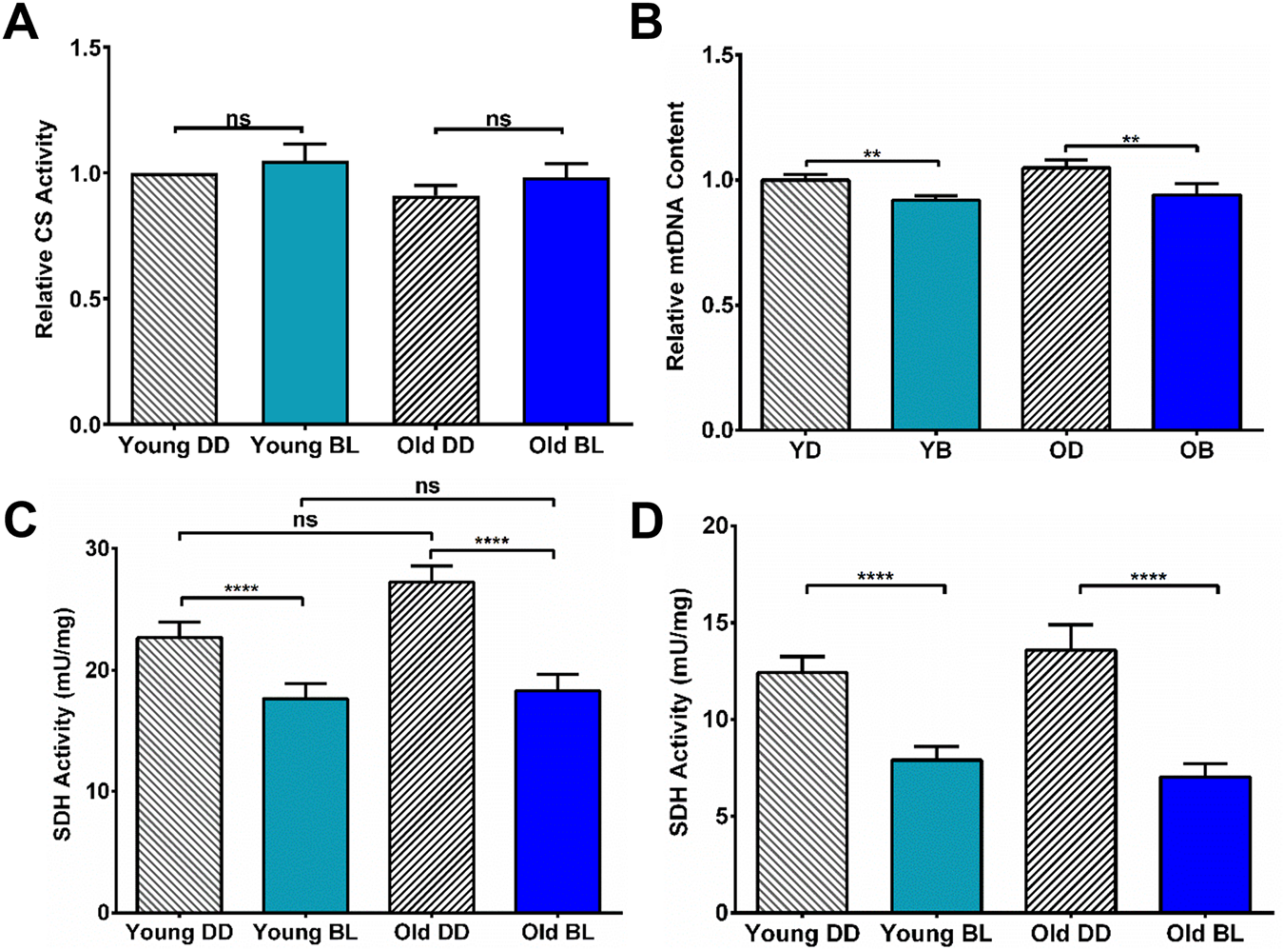
Biochemical characterization of mitochondria after BL and aging. **A** Citrate synthase (CS) activity did not differ significantly between samples representing different ages and light conditions. *N* = 5 independent biorepeats. Values are normalized to citrate synthase activity in young DD flies set as 1 for each bio-repeat. **B** Relative mtDNA content did not decline with age in DD flies while it was modestly reduced in BL-exposed flies. *N* = 15 individual flies for each conditions. **C** Activity of succinate dehydrogenase (SDH) measured in whole flies was not significantly altered by age in DD flies, but was significantly reduced in both young and old flies exposed to BL for 10 days. **D** Blue light exposure also reduced SDH activity in isolated heads of both young and aging fly brains. Statistical analysis by 2-way ANOVA (**p < 0.01; ****p < 0.0001). Error bars indicate SEM.

One of the reasons for the decreased respiration rates in mitochondria from BL-exposed flies could be a reduced number of mitochondria in these flies. To compare mitochondrial content between experimental conditions, we first measured the maximum activity of citrate synthase in aliquots of the mitochondria samples used for respirometry. As seen in Fig. 4A, citrate synthase activity did not differ significantly between samples representing different light conditions and ages. In addition, we measured mitochondrial DNA (mtDNA) copy number as it is a critical component of overall mitochondrial health^12^. Using quantitative PCR, we measured ratio of mtDNA and nuclear DNA (nucDNA) and found that the relative mitochondria content did not significantly change with age (Fig. 4B). However, it was modestly but significantly reduced (*p* < 0.01) in both young and old flies exposed to BL for 10 days compared to age-matched DD controls. Taken together, these data suggest that overall mitochondria density does not decline with age but is somewhat reduced by blue light treatment.

Given that BL significantly reduced respiration of succinate dehydrogenase (SDH)-linked CII (Fig. 3), we next measured the activity of SDH in fly homogenates using a microplate assay. SDH activity was similar between young and old flies kept in DD. In contrast, SDH activity was significantly reduced (*p* < 0.001) in both young and old flies exposed to BL for 10 days compared to age-matched DD counterparts (Fig. 4C). Additionally, we measured SDH activity in isolated heads and determined that SDH activity was decreased by BL exposure (Fig. 4D) even more dramatically than in whole flies. SDH activity was reduced in whole flies by 24% in young and 32% in old, while in the heads it was reduced by 36% and 48% in young and old, respectively.

We also tested whether age and/or BL treatment affected the activity of Complex IV (CIV) by measuring the oxidation rate of reduced cytochrome C in a microplate assay. The activity of Cytochrome C oxidase (COX) did not change significantly in young or old flies exposed to BL compared to DD controls. However, COX activity was significantly lower in old flies compared to young ones (*p* < 0.001) irrespective of light treatment (Supplementary Fig. 1).

**Fig. 5:**
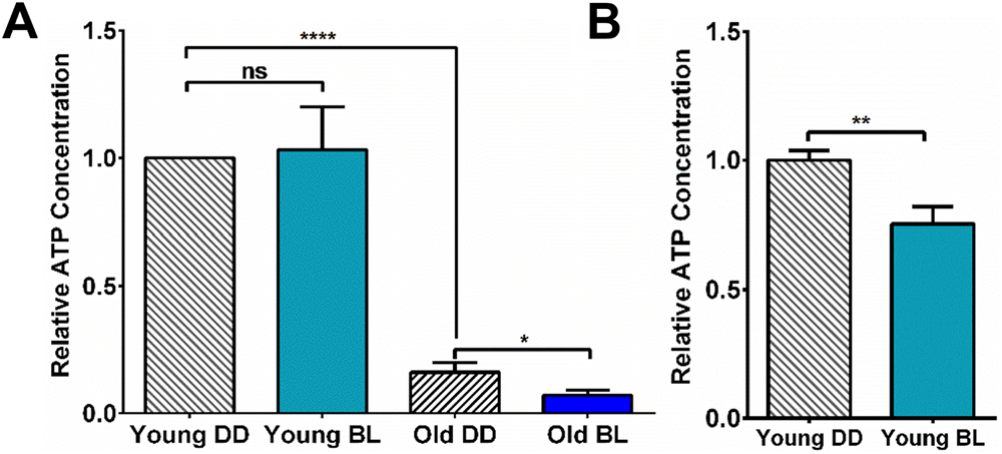
ATP levels are dramatically affected by age and moderately by BL exposure. **A** ATP levels in young and old DD and BL whole flies. **B** ATP levels in isolated heads of young DD and BL flies. *N* = 5 independent biorepeats of 25 flies for each conditions. Values are normalized to ATP levels in young DD flies set as 1 for each bio-repeat. Statistical analysis by *t* test (*p < 0.05;**p < 0.01; ****p < 0.0001). Error bars indicate SEM.

The decrease in activity of various mitochondrial enzymes with age and BL treatment would be expected to compromise energy production. Therefore, we measured steady-state levels of ATP as a function of BL exposure and age using bioluminescent assays. While ATP content was not significantly affected by BL in young whole flies, a dramatic decline in ATP levels was recorded in old flies kept in DD (*p* < 0.001) and father reduction (*p* < 0.05) was seen in old BL flies (Fig. 5A). In addition, significantly reduced ATP was detected in isolated heads of young BL flies (Fig. 5B). These data suggest that substantial decline in mitochondrial energy production occurs in *Drosophila* during aging and further decline is caused by BL exposure, especially in fly heads.

Given the impairment in mitochondrial function and previous reports that BL exposure generates ROS in fly photoreceptors^4^ and in murine retina-derived cells in vitro^6^, we sought to determine whether BL may cause increased ROS levels in our flies. Levels of hydrogen peroxide were measured in isolated mitochondria using Amplex Red microplate assay. For a positive control, we kept flies for 5 days in hyperoxia (100% O_2_ under atmospheric pressure) as described^13^; this verified that our protocol could detect H_2_O_2_ increase (Supplementary Fig. 2). We then measured H_2_O_2_ in mitochondria from young and old flies kept in BL or DD for 10 days and did not detect significant differences between any of the groups (Supplementary Fig. 2). Given that the nervous system is more likely to encounter oxidative stress than other tissues, we also measured ROS in fly brains using an anti-nitrotyrosine antibody. Interactions with increased levels of reactive oxygen and nitrogen species cause irreversible nitration of tyrosine in cellular proteins and this is used as a biomarker of accumulated oxidative stress^14,15^. As positive control, isolated brains were incubated with peroxynitrite as described^14^, which causes instant tyrosine nitration. This treatment caused a dramatic increase in fluorescence (Supplementary Fig. 3). However, exposure of young or old flies to BL for 10 days did not visibly increase brain fluorescence compared to DD controls (Supplementary Fig. 3). While we were not able to detect ROS in tissues of *eya* flies exposed to BL, we also tested whether BL-exposed flies may be more susceptible to exogenous oxidative stress in the form of hyperoxia environment (100% oxygen)^13^. We observed dramatically reduced survival in young flies that were kept in BL for 6 or 10 days and then exposed to oxidative stress compared with flies kept in DD before hyperoxia test (Supplementary Fig. 4).

## Discussion

Understanding the effects of BL on cellular functions is becoming a significant health issue as humans are exposed to more blue-enriched LED illumination for most of the day and even at night due to shift work and light pollution in large cities^1^. While acute BL is phototoxic to different cells types in vitro^16^, it is not known whether daily BL exposure across the human lifespan may have long-term effects on cellular functions. Research on model organisms could provide insights into the effects of BL on conserved cellular pathways. Having previously reported that daily exposure to 12 h of BL accelerates aging in adult *Drosophila*^8^, we investigated the effects of constant BL on young, middle age, and old flies. We demonstrate that BL affects flies differently across their lifespan with decreased survival and increased brain neurodegeneration observed in old flies after the same length of BL exposure. Furthermore, our data suggest that age-dependent impairments in mitochondrial respiration and energy production contribute to the increased phototoxic effects of BL in older flies.

There are numerous reports on toxic effects of acute BL on photoreceptors in mammals and flies as well as in retina-derived cells exposed to BL in vitro^3–6,17^; yet, studies on the effects of chronic BL at the organismal level are sparse. Visible light of different wavelengths shortened the lifespan of the nematode *C. elegans*, with BL showing the strongest effects^7^. Further, BL (but not other wavelengths) reduced the lifespan in *Drosophila*^8,18^, but the cellular mechanisms underlying these effects are not understood and it is not known whether BL responses are affected by organismal aging. To address these questions, flies in the current study were aged in DD and then exposed to constant BL at different ages. The results show that fly lifespan was shortened proportionally to the age at which the flies were transferred to BL conditions. We also determined that 10 days of BL exposure at young or middle age affect subsequent longevity in DD. We found that even exposure of young flies to 10 days of BL shortened their subsequent survival in DD, and exposure at middle age shortened their lifespan even more significantly. Thus, the phototoxic effects persist after removing of the BL stressor and their severity depends on the age at which flies were in BL. We confirmed that these effects are specific to blue light, since flies exposed to constant white light with blue wavelengths blocked did not show reduced lifespan.

Our data show that decreased survival of old flies in BL is associated with increased brain neurodegeneration. Young flies kept in darkness showed negligible brain vacuolization that did not increase significantly after 10 days in BL. Old flies aged in darkness displayed some brain vacuolization; however, it was significantly increased when aging flies were exposed to 10 days in BL. Together, these data show that BL is an environmental stressor that impacts organisms more severely with age, similar as was shown for starvation and oxidizing agents in organisms from flies to mammals^19,20^.

To explore the reasons for reduced survival and increased neurodegeneration in old flies exposed to BL, we investigated mitochondrial functions. We first measured mitochondria content as a function of age and BL exposure. Citrate synthase activity, which is used to estimate mitochondria levels remained similar with age and BL treatment. More direct measurement of mtDNA show no difference with age; however, mtDNA was reduced in both young and old BL-exposed flies suggesting that lower counts of mitochondria may contribute to detrimental effects of BL. Secondly, we measured the impact of BL and age on oxidative phosphorylation and determined that it varied between mitochondrial complexes. The activity of CI was more significantly reduced by age than by light. On the other hand, the activity of CII was dramatically reduced by BL exposure and less affected by age. Damaging effects of BL on CII were further confirmed by biochemical assays that showed significantly decreased activity of SDH in whole flies; reduction of SDH activity was even more pronounced in isolated heads suggesting that mitochondria impairments could be linked to neurodegeneration. In contrast to CII, G3P-stimulated respiration through CIII was not affected by either BL or aging. Finally, we found that the activity of CIV was significantly lower in old flies only, both in DD and BL. Likewise, ATP content was dramatically reduced by age and less affected by BL; however, we detected a significant reduction in ATP levels in heads of young flies that were exposed to BL for 10 days. While this study appears to be the first to report the effects of BL on oxidative phosphorylation, our data on the effects of age on enzyme activities are in agreement with previous reports that the activity of CI and IV as well as ATP levels declined with age in flies, while the activity of CII remained unchanged^21,22^.

One of the novel findings of our study is that CII (succinate dehydrogenase) is negatively affected by BL in *Drosophila* and this is independent of age. CII functions in both the tricarboxylic acid cycle (TCA cycle) and the electron transfer system, and dysfunctions of SDH are linked to cancer development^23^ and neurodegeneration^24^. While the mechanism behind specific BL action on SDH activity remains to be investigated, SDHA, the catalytic subunit of SDH complex requires the flavin adenine dinucleotide (FAD) cofactor, which is derived from riboflavin. Riboflavin is maximally activated by BL of 450 nm, close to the peak of 460 nm, which was used in our study and is also prevalent in LEDs due to the low cost of manufacturing^25^. Since BL can degrade riboflavin into non-active photoproducts^26^, we speculate that BL could impair the activity of SDHA by affecting its flavination status. However, we note that G3P dehydrogenase is also a FAD-dependent enzyme, yet, it was not affected in our experiments, so other, as yet unknown, factors may contribute to SDH impairment.

**Fig. 6:**
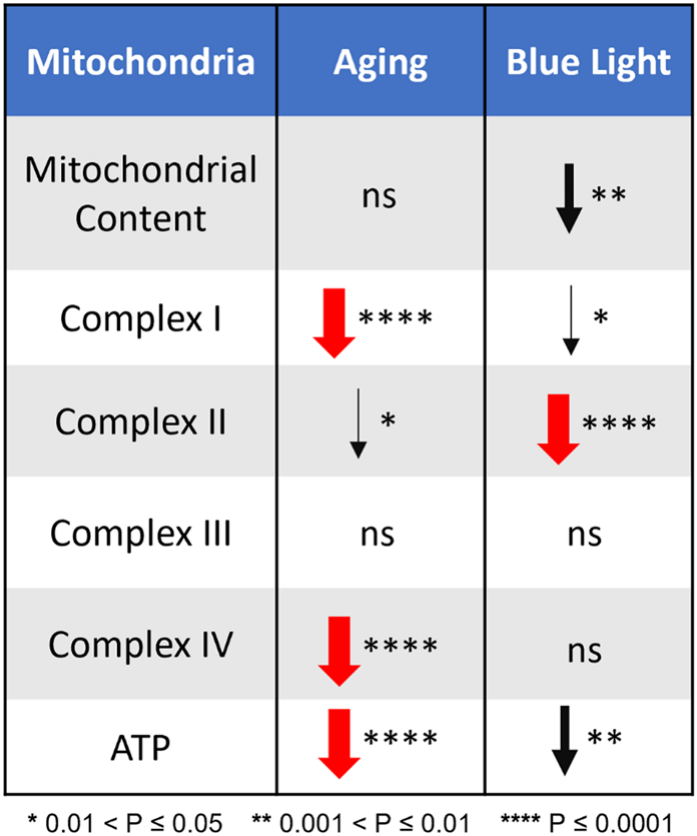
Schematic summary of results showing how respiratory functions of mitochondria are reduced by aging and BL exposure, respectively. Statistical significance is represented by the thickness of the arrow. Non-significant changes are marked as “ns”.

Our study implies that BL-induced decline in CII activity is less detrimental in young flies because other mitochondrial complexes are not affected by BL, and ATP levels are only slightly reduced. In contrast, BL-induced decline in CII respiration in old flies occurs on top of significant impairment of CI and IV and dramatically reduced ATP levels (Fig. 6). We propose that this situation leads to increased mortality and neurodegeneration in BL-exposed aging flies compared to young ones. It is possible that BL also impairs other cellular processes not investigated in our study, such as loss of protective mechanisms and/or accumulation of damaged proteins during aging.

The damaging effects of excessive light on photoreceptive cells in the retina of flies and mice are well documented^27^. These cells are specialized in light detection and their greatly folded membranes are packed with rhodopsin and other proteins involved in phototransduction. Excessive light exposure appears to disrupt rhodopsin trafficking and cause lipid peroxidation contributing to cell death^4,28^. Most studies on BL effects also report an increase in cellular ROS in retina-related cells in vitro^5,6^. In contrast, we were not able to detect increased ROS levels measured in mitochondria isolated from young or old *eya* flies or in their brains. Nevertheless, we found that BL-exposed flies are less resistant to exogenous oxidative stress as their survival in hyperoxia was significantly reduced compared to DD controls. This provides further evidence that BL acts as pro-aging factor in flies.

Our study was conducted in flies with genetically ablated eyes suggesting that BL affects extra-retinal cells, including neurons in the brain. Indeed, there is increasing evidence that cells that are not specialized in visual photoreception are also sensitive to BL. For example, extended exposure to blue (but not green or red) light resulted in increased expression of neuronal activity-regulated genes in cortical neurons in vitro^29^. Further, studies of human fibroblasts in vitro and in vivo show that BL at different wavelengths and intensity may induce varying degrees of oxidative stress and inhibit proliferation, which could contribute to premature skin photo-aging^16,30^. Of note, it was reported that BL can penetrate human skin to regulate physiology of subcutaneous white adipocytes^31^.

In summary, our results show that damaging effects of BL at the organismal level are strongly age dependent and are associated with reduced activity of specific components of energy-producing pathways in mitochondria.

## Methods

### Drosophila rearing, light exposure, longevity, and neurodegeneration

*Drosophila melanogaster* was maintained on diet containing yeast (35 g/l), cornmeal (50 g/l), and molasses (5%) at 25 ± 1 °C. Wild-type Canton S (CS) flies were used in the initial experiments, but most experiments were performed on eyes absent (*eya*^*2*^) mutants that do not develop compound eyes^9^. Flies used in the experiments were mated and separated by sex when 1-2 days old. Fly colonies were reared in cycles of 12 h of fluorescent light alternating with 12 h darkness. Experimental adult flies were maintained in constant darkness or constant blue light with a peak emission of 460 nm produced by the MarsAqua Dimmable 165 W LED Light with a photon flux density of 20–30 µmol/m^2^/sec (irradiance of ~0.4 mW/cm^2^) measured at the level of horizontally placed narrow vials (Genesee Scientific), each containing 25 flies. For the experiment excluding blue light from the light source, flies were exposed to white LEDs with yellow long-pass filter giving peak emission of 584 nm and irradiance of~0.4 mW/cm^2^, as described^8^.

For each genotype and light condition, lifespan was measured using at least 50 males or females held in groups of 25 with mortality recorded and fresh diet provided every 2-3 days. Mortality curves were statistically analyzed using the Log-rank test in GraphPad Prism 6.

To quantify light-induced neurodegeneration in the brain, we measured the area of all vacuoles seen on sections of the brain as described previously^32^. In addition, we counted vacuoles in the antennal lobes. Analyses were done double-blind and statistical significance was determined with unpaired t-tests using GraphPad Prism 6.

### Mitochondrial respiration

Mitochondria were obtained from whole flies then analyzed using high-resolution respirometry (Oxygraph O2K; Oroboros Instruments, Innsbruck, Austria). Flies were homogenized and respiration was conducted in MiR05 respiration buffer (0.5 mM EGTA, 3 mM MgCl2-6H2O, 60 mM lactobionic acid, 20 mM taurine, 10 mM KH2 PO4, 20 mM HEPES, 110 mM sucrose, and 1 g/L bovine serum albumin). For each sample, 50 males were gently homogenized by hand on ice in glass-on-glass homogenizers with 0.3 mm spacing between mortar and pestle for 2 min. Samples were centrifuged for 10 min at 1,000 *g* at 4 °C. Mitochondria-enriched supernatant was removed and 200 µl was injected into a chamber of Oxygraph-2k for high-resolution respirometry. Instrument settings were 25 °C, stirring at 750 RPM and 2-s data averaging (Datlab 6, Oroboros Instruments). Oxygen consumption was measured after the addition of mitochondrial suspension; then substrates and reagents were added (as indicated in the “Results”) in the following concentrations: 10 mM glutamate, 2 mM malate, 5 mM pyruvate, 2.5 mM ADP, 10 mM succinate, 0.5 μM rotenone, 10 mM G3P (glycerol-3-phosphate), and 2.5 mM antimycin A (AA). The protein concentration of the mitochondrial preparation was measured using Qubit Protein Assay Kits (Invitrogen). Oxygen flux was calculated relative to protein content (pmol O_2_/mg mitochondrial protein/s).

### Citrate synthase assay

Citrate syntase activity was measured in aliquots of mitochondria that were prepared for O_2_ flux (see above), diluted 10 times and added to the reaction mixture. The activity was measured as the increase in absorbance due to the reduction of 5,5′-dithiobis-2-nitrobenzoic acid (DTNB) at 412 nm, coupled to the reduction of acetyl-CoA by citrate synthase in the presence of oxaloacetate. The reaction mixture contained 88 mM Tris-HCl pH 8.1, 300 µM acetyl-CoA, 0.5 mM oxaloacetate, 100 µM DTNB. The absorbance was immediately read at 412 nm in kinetic mode for 6 min at 25 °C using a BioTek Synergy 2 microplate reader. The citrate synthase activity was calculated as described^33^ and normalized to the activity in young flies in dark conditions set as 1.

### Succinate dehydrogenase (SDH) assay

SDH activity was measured using the Succinate Dehydrogenase Activity Colorimetric Assay Kit (Sigma-Aldrich) according to the manufacturer’s instructions. For each sample, 4 frozen whole male flies or 25 isolated heads were rapidly homogenized in a 1.7 ml Eppendorf tube with 100 µl of ice-cold SDH assay buffer using a motorized pestle homogenizer (Kontes), kept on ice for 10 min, and centrifuged at 10,000 *g* for 5 min at 4 °C. The supernatant was transferred to a fresh tube. In a clear 96-well plate, 10 µl of supernatant was mixed with 92 µl of reaction mix (88 µl of SDH assay buffer, 2 µl of SDH substrate, and 2 µl of SDH probe) in each well. The absorbance was immediately read at 600 nm in kinetic mode for 30 min at 25 °C using a BioTek Synergy 2 microplate reader. The SDH activity was calculated according to the manufacturer’s instructions and normalized to total protein concentration of the sample.

### Cytochrome C oxidase (COX) assay

COX activity was measured using the Cytochrome C Oxidase Assay Kit (Abcam) according to the manufacturer’s instructions. The samples were processed as described for the SDH assay and the supernatant was diluted 20 times with Enzyme Dilution Buffer. In a clear 96-well plate, 5 µl of sample was mixed with 120 µl of reaction mix (100 µl of Assay Buffer and 20 µl reduced cytochrome c) in each well. The absorbance was immediately read at 550 nm in kinetic mode for 30 min at 25 °C using a BioTek Synergy 2 microplate reader. The COX activity was calculated according to the manufacturer’s instructions and normalized to total protein concentration of the sample.

### Determination of mitochondria DNA (mtDNA) copy number

PCR method was used to measure mtDNA relative to nucDNA as described^12^. For each sample, single fly was homogenized in a 1.7 ml Eppendorf tube with 50 µl of ice-cold buffer (10 mM Tris-HCl, 25 mM sodium chloride, 1 mM EDTA, and 200 µg/ml proteinase K, pH 8.2) using a motorized pestle homogenizer (Kontes). Samples were kept on ice followed by incubation at 37 °C for 20 min. After incubation, the tubes were placed in heat block at 95 °C for 1.5 min. then centrifuged at 14,000 *g* for 2 min; supernatants were transferred to fresh tubes and re-centrifuged to obtain clean genomic DNA. The DNA samples were diluted 1–50 using nuclease free water for qPCR. Each PCR reaction contained 3 µl of diluted genomic DNA, 1 µl of mtDNA or nucDNA target specific primer pair (400 nM final concentration each), 12.5 µl SYBR Green PCR Master Mix (Applied Biosystems) and 8.5 µl H_2_O. Quantitative PCR was performed in a Quant Studio 3 (Applied Biosystems) running the standard program. The mtDNA content relative to nucDNA was determined as follows: (1) ∆CT = nucDNA CT—mtDNA CT; (2) Relative mtDNA content = 2 × 2∆CT. qPCR primer sequences: (1) mtDNA (151 bp) forward—GCTCCTGATATAGCATTCCCACGA; reverse—CATGAGCAATTCCAGCGGATAAA. (2) nucDNA (152 bp) forward—CGAGGGATACCTGTGAGCAGCTT; reverse—GTCACTTCTTGTGCTGCCATCGT.

### ATP assay

For each sample, 25 males or 25 male heads were homogenized in a chilled glass homogenizer manually in 400 or 200 µl MiR05, respectively, on ice for 2 min. The samples were centrifuged at 1000*g* for 10 min at 4 °C. For each sample, 60 µl of mitochondrial extract was aliquoted to a fresh tube and boiled immediately for 10 min, then centrifuged at 20,000 *g* for 5 min at 4 °C. Then the supernatant was transferred to a fresh tube. The steady-state ATP content was measured using the ATPlite Luminescence ATP Detection Assay System (Perkin Elmer). In a white 96-well plate, 10 µl of sample was mixed with 90 µl of Schneider’s medium (Gibco, 21720-024) and 50 µl Cell Lysis Solution in each well. Then 50 µl of substrate solution was added to each sample well and mixed by shaking the plate for 5 min on an orbital shaker. The plate was dark adapted for 10 min and the luminescence was measured at 25 °C using a BioTek Synergy 2 microplate reader. The ATP level was calculated using ATP standard curve and normalized to protein concentration of the sample.

## Supplementary information

Supplementary

## Data Availability

All data generated or analyzed during this study are included in this published article (and its Supplementary information files). Any additional material are available from the corresponding author. All data are available upon request.
