# Supplementary material for: Age-dependent effects of blue light exposure on lifespan, neurodegeneration, and mitochondria physiology in Drosophila melanogaster
Source: NPJ Aging. 2022 Jul 27;8(1):11. doi: 10.1038/s41514-022-00092-z (PMC9329351; doi:10.1038/s41514-022-00092-z)
Supplement: Supplementary file 1 — Supplementary [file 41514_2022_92_MOESM1_ESM.pdf]

SUPPLEMENTARY MATERIAL

Song et al., Age-dependent effects of blue light exposure on lifespan, neurodegeneration, and mitochondria physiology in Drosophila melanogaster.

SUPPLEMENTARY FIGURES

Supplementary Figure 1. Activity of Cytochrome C oxidase is reduced by age independent of light exposure.

Supplementary Figure 2. Blue light exposure does not increase hydrogen peroxide production in isolated mitochondria.

Supplementary Figure 3. Blue light exposure does not increase levels of ROS marker, nitrotyrosine, in fly brains.

Supplementary Figure 4. BL-exposure increase susceptibility of flies to oxidative stress.

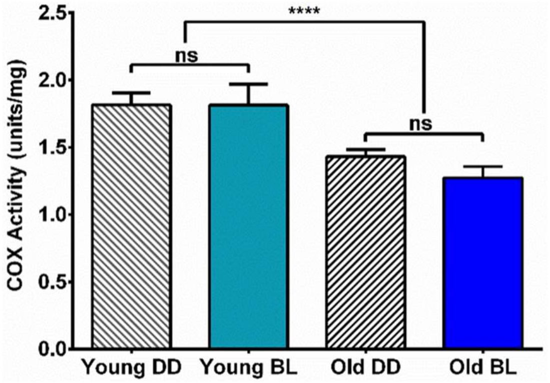

Supplementary Figure 1. Activity of Cytochrome C oxidase (COX) was significantly reduced with age. N= 5 independent biorepeats. Statistical analysis by 2-way ANOVA, (****$p<0.0001$). Error bars indicate SEM.

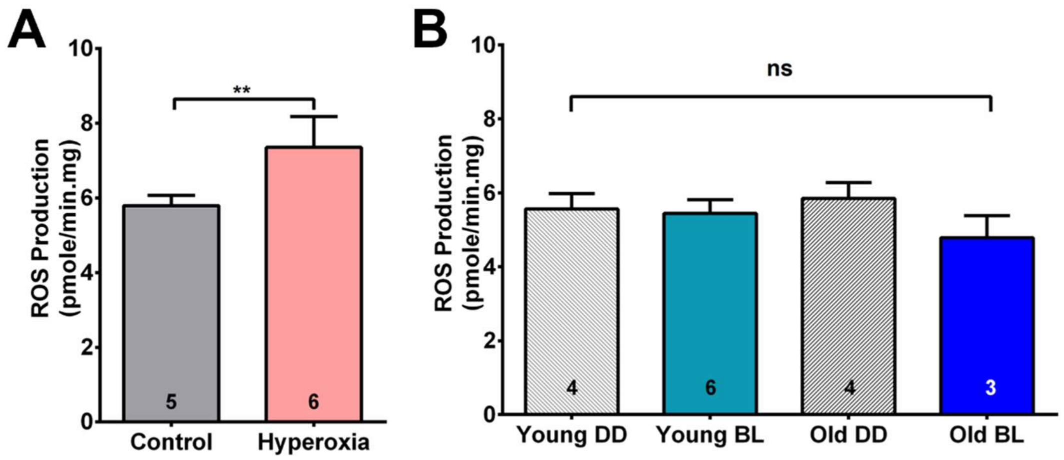

Supplementary Figure 2. Blue light exposure does not increase hydrogen peroxide (ROS) production in isolated mitochondria. (A) *eya*^{2} males kept in hyperoxia environment with 12 hours regular light and 12 hours dark showed significantly increased ROS production compared to flies in normoxia. (B) ROS production did not differ significantly between samples representing different ages and light conditions. Number of biorepeats indicated inside bars. Statistical analysis by unpaired T-test, (** $p<0.01$). Error bars indicate SEM.

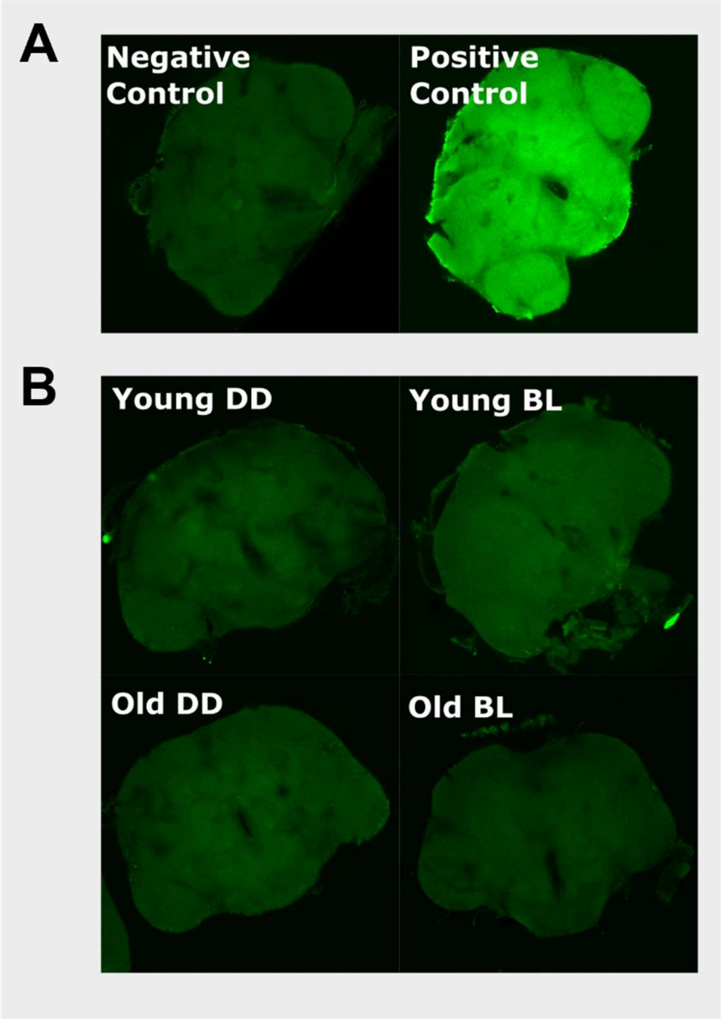

Supplementary Figure 3. Blue light exposure does not increase levels of ROS marker, nitrotyrosine, in fly brains. (A) Anti-nitrotyrosine labeled immunofluorescence demonstrated increased expression of ROS marker, nitrotyrosine, in brains treated with peroxynitrite (Positive Control) to maximize the amount of nitrotyrosine. Negative control represents brains in which primary antibody was omitted. (B) Nitrotyrosine levels did not differ significantly between samples representing different ages and light conditions with the same exposure time. N= 5 brains for each treatment.

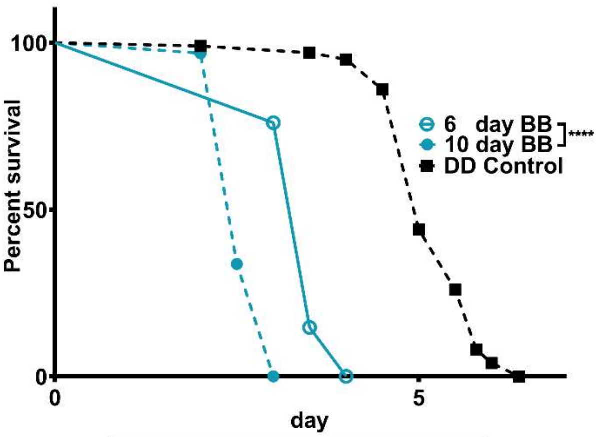

	6 day BB	10 day BB	DD
Median survival	3.5	2.5	5

Supplementary Figure 4. BL-exposure increases the susceptibility of flies to oxidative stress. Young flies that were kept in BL or DD for 6 or 10 days were subsequently transferred to hyperoxia conditions. Two biological replicates of 50 flies each were conducted and mortality curves were analyzed by log by log-rank test. Statistics for mortality curves by log-rank test (****p<0.0001).

SUPPLEMENTARY METHODS

ROS Measurement in isolated mitochondria. Fifty males were homogenized manually in a chilled glass homogenizer in 500 µl of mitochondrial isolation medium (MIM: 250 mM sucrose, 10 mM Tris-HCl pH 7.4, 0.15 mM MgCl2) on ice for 2 min. The sample was transferred to an Eppendorf and centrifuged at 1,000 g for 5 min at 4 °C. The supernatant was transferred to a fresh tube and centrifuged at 12,000 g for 5 min at 4 °C to obtain mitochondria enriched pellets. The pellet was washed with 1 ml of MIM, re-centrifuged at 7,000 g for 5 min at 4 °C, and resuspended in 50 µl of MIM. H_20_2 levels were measured using Amplex Red Hydrogen Peroxide Assay Kit (Invitrogen) following manufacturer's directions. In a clear 96-well plate, 20 µl of mitochondria solution was mixed with 30 µl of 1 x reaction buffer and 50 µl of working solution (48.5 µl of 1 x reaction buffer, 1 µl of horseradish peroxidase, and 0.5 µl of Amplex red solution) in each well. The plate was protected from light and incubated on an orbital shaker at room temperature (RT) for 3h. The fluorescence was measured at 25 °C using a BioTek Synergy 2 microplate reader. The hydrogen peroxide production was calculated by using H_20_2 standard curve and normalized to total protein concentration of the sample.

Nitrotyrosine detection by Immunofluorescence. Flies were fixed with 4% paraformaldehyde in (Phosphate-Buffered Saline (PBS) with 0.1% Triton X-100 at RT for 3 hours then washed with PBS with 0.5% Triton X-100 (PBST) 3 times for 10 minutes. Brains were dissected in PBS. For a positive control, 5 brains were incubated in 1 mM peroxynitrite (ONOO-) in PBS for 5 min to produce nitrotyrosine. All brains were then incubated in PBST with 5% normal goat serum (NGS) for 3 h, followed by primary rabbit antibody (anti-tyrosine nitrate, 1:200) for 1 hour at RT then overnight at 4 °C. Some brains were kept in PBS without primary antibodies as the negative control. All brains were washed in PBST 5 times 15 min at RT and incubated with secondary antibody (goat anti-rabbit Alexa 488, 1:1000) for 1 hour at RT and overnight at 4 °C. Next, brains were washed 4 times 30 min in PBST and once in PBS with 0.1% Triton X-100. Brains were mounted in Vectashield with DAPI nuclear stain and imaged with a Zeiss LSM 780 NLO scanning confocal microscope (Zeiss) with all laser parameters set for optimal signal in positive control and then held constant while imaging young and old BL and DD flies.

Exposure of flies to hyperoxia. To test survival of BL-exposed flies to oxidative stress, we used hyperoxia (HO). Young flies that were kept in BL or DD for 6 or 10 days were subsequently transferred to clear, airtight chambers with 100% O2 flow-through at atmospheric pressure placed in dark room. Mortality of flies was recorded every 12 h. Two biological replicates of 50 flies each were conducted and mortality curves were analyzed by log by log-rank test.
